# Natural Bioactive Compounds: Integrated Analytical and Biological Approaches for the Special Issue “Analysis and Biological Evaluation of Bioactive Compounds from Natural Sources”

**DOI:** 10.3390/molecules31091385

**Published:** 2026-04-22

**Authors:** Luana Pulvirenti, Rosanna Avola

**Affiliations:** 1 Istituto di Chimica Biomolecolare del CNR, Via P. Gaifami, 18, 95126 Catania, Italy; 2 Department of Medicine and Surgery, Kore University Enna, 94100 Enna, Italy

## 1. Introduction

The investigation of natural bioactive compounds represents a cornerstone of contemporary scientific research, with profound implications for clinical medicine, nutraceuticals, cosmetics, and biotechnology. The extraordinary biodiversity of plant, fungal, and marine ecosystems provides a vast reservoir of structurally diverse secondary metabolites—including polyphenols, flavonoids, alkaloids, terpenes, and bioactive peptides—characterized by remarkable chemical complexity and broad pharmacological potential [1,2,3,4].

About 40% of modern drugs currently in clinical use derive from natural compounds or their semisynthetic derivatives, with particular emphasis on the classes of antineoplastics (paclitaxel, vinblastine), antimicrobials (erythromycin, tetracyclines) and immunomodulators (artemisinin, cyclosporine) [5]. Over the past four decades, natural products have significantly contributed to drug discovery, accounting for a substantial proportion of newly approved therapeutic agents [6].

This Special Issue, “Analysis and Biological Evaluation of Bioactive Compounds from Natural Sources”, showcases cutting-edge contributions that exemplify this continued evolution. Driven by rising consumer awareness, the global natural nutraceutical market is projected to grow from USD 117 billion in 2023 to USD 650 billion by 2032, at a CAGR of 17.1% [7].

In the current scientific landscape, however, simple extraction and structural identification are no longer sufficient. Phytochemical characterization must be complemented by rigorous biological validation to elucidate mechanisms of action, define molecular targets, and evaluate safety and efficacy profiles [2,3,4]. This paradigm shift reflects the growing need for an integrated research strategy capable of translating natural diversity into clinically and industrially relevant applications.

Such an approach relies on a multidisciplinary framework that combines analytical chemistry, metabolomics, molecular biology, pharmacology, and biotechnology. Advanced analytical tools, including high-performance liquid chromatography (HPLC), high-resolution mass spectrometry, nuclear magnetic resonance (NMR), and metabolomic profiling, enable the precise characterization of complex natural matrices [7,8,9]. For instance, MALDI-TOF imaging has successfully mapped 42 secondary metabolites in pomegranate peel, identifying bioactive ellagitannins with anticancer potential [10]. These technologies allow the detection of trace-level compounds, structural elucidation of novel metabolites, and comprehensive chemical fingerprinting, thereby accelerating the discovery process.

Simultaneously, *in vitro* and *in vivo* experimental models are essential for the functional validation of antioxidant, anti-inflammatory, antimicrobial, antidiabetic, neuroprotective, and anticancer activities [2,11]. The integration of chemical profiling with mechanistic biological studies strengthens the translational potential of natural compounds and supports their progression toward clinical and technological applications.

Sustainability has become an equally important dimension of natural product research. Environmentally friendly extraction methodologies are increasingly adopted to reduce solvent consumption and ecological impact, aligning research practices with circular economy principles [3,12]. In this context, the valorization of agro-industrial by-products represents a promising strategy to recover high-value bioactive molecules from waste materials, improving resource efficiency and environmental sustainability [12].

Enzymatic extraction has enabled the recovery of high-value pectin from the approximately 15 million tons of orange peel waste generated annually [13]. Recent investigations further highlight the translational relevance of this integrated approach. Cannabigerolic acid (CBGA), for example, has demonstrated the ability to modulate enzymatic and cellular pathways involved in oncological, metabolic, and inflammatory disorders, suggesting promising therapeutic perspectives [14].

Research on phytochemical characterization and biological validation also illustrates the multidimensional nature of this field. Studies on blood oranges have demonstrated that rootstock selection significantly influences metabolite composition and antioxidant capacity, emphasizing the interplay between agronomic variables and nutraceutical quality [15]. Investigations into the antidiabetic potential of vanadium complexes combined with olive leaf extracts have proposed innovative strategies to enhance therapeutic efficacy while mitigating metal-associated toxicity [16]. Similarly, the recovery of hypoglycemic compounds from industrial distillation wastewater of *Lamiaceae* species has underscored the technological and environmental value of waste valorization strategies [17].

At the biomedical level, natural compounds continue to demonstrate compelling therapeutic potential. Essential oils from *Fabiana imbricata* have been shown to induce apoptosis in prostate cancer cells through reactive oxygen species modulation [18], while pholiotic acid exhibits significant pro-apoptotic activity in metastatic melanoma models [19]. Furthermore, increasing evidence supports the role of essential oils as modulators of neuroinflammation, opening promising perspectives for the management of neurodegenerative diseases [20].

Ibrahim et al. (contribution 1) reported, among other examples, the use of chitosan–oleic acid nanoparticles with lemon peel essential oil for topical treatment of vulvovaginal candidiasis, whereas Guo et al. (contribution 2) investigated the hepatoprotective and therapeutic potential of emodin in liver fibrosis.

Beyond biomedical research, technological implications extend to pharmaceutical production systems and agricultural innovation. Plant-based production platforms and genome-editing technologies such as CRISPR are reshaping the sustainable generation of optimized bioactive molecules [21]. In agriculture, natural bioactive compounds are increasingly employed as eco-compatible biopesticides and biostimulants, contributing to crop protection while minimizing environmental impact [22]. White biotechnology approaches further enable the sustainable conversion of renewable biomass into valuable functional ingredients [22].

The scientific and applicative relevance of these research directions is reinforced by the growing global demand for safe and effective natural products, both as therapeutic agents and as functional ingredients in food and cosmetic formulations. Market expansion is driven by increasingly informed consumers and regulatory frameworks encouraging low-impact and environmentally sustainable substances. In this scenario, advanced analytical characterization combined with rigorous biological evaluation serves as a fundamental bridge between basic research and industrial implementation, enabling the transformation of biodiversity into tangible resources for health, well-being, and sustainable development [23,24]. Liposomal curcuminoids increase the bioavailability of turmeric by 185 times compared to the free form [25].

## 2. Advancement of Analytical Methodologies

A key objective in natural product research is the development and refinement of analytical methodologies capable of identifying and structurally characterizing bioactive compounds within complex matrices. The inherent chemical diversity and low abundance of many secondary metabolites demand highly sensitive, selective, and reproducible techniques. Recent studies by Kamierszczak et al. and Li et al. (contributions 3 and 4) highlight how modern platforms, such as high-performance liquid chromatography (HPLC) coupled with high-resolution mass spectrometry (HRMS) and next-generation nuclear magnetic resonance (NMR), now allow comprehensive metabolite profiling even in highly complex biological systems. The LC-HRMS/MS platform combined with metabolomics identified 127 polyphenols in Sicilian olives, related to varietal resistance [26]. These approaches enable accurate structural elucidation, quantitative analysis, and metabolomic fingerprinting, accelerating the discovery of compounds with potential therapeutic and industrial applications.

Equally important is the integration of sustainable extraction strategies and optimized sample preparation protocols. Taneva et al. (contribution 5) described green extraction technologies, including ultrasound-assisted extraction, supercritical fluid extraction, and solvent-minimization techniques, that enhance the recovery of bioactive compounds while reducing environmental impact [27]. Ultrasound-assisted extraction increased anthocyanin recovery from blueberry residues by 40%, while preserving antioxidant activity [28]. By combining environmentally responsible extraction methods with advanced analytical instrumentation, researchers can achieve reproducible, scalable, and biologically relevant results that align with the principles of sustainability and circular economy models. The integration of AI with GC-MS predicted with 92% accuracy the flavor profile of essential oils from Sicilian wild plants [29].

## 3. Extraction Methodologies and Chemical Characterization

Recent studies emphasize the importance of refining extraction procedures alongside detailed chemical profiling to maximize both the yield and quality of bioactive compounds. For example, solvent-assisted extraction methods have been systematically optimized to improve polyphenol recovery from plant materials. Qi et al. (contribution 6) reported adjusting solvent polarity and extraction duration allows not only higher total phenolic content but also preservation of antioxidant activity, as confirmed by HPLC-DAD and radical scavenging assays such as DPPH and ABTS. Extraction using natural deep eutectic solvents (NADESs) recovered 92% of polyphenols from olive residues, demonstrating negligible toxicity [30]. These improvements demonstrate that extraction efficiency can be enhanced without compromising compound integrity, providing a strong foundation for subsequent functional studies.

In addition to polyphenols, lipid-soluble bioactives have been thoroughly investigated. Detailed fractionation and chromatographic analysis of *Amsonia tabernaemontana* seed oil enabled quantification of sterols, tocopherols, phospholipids, and fatty acids, including minor unsaponifiable components like β-sitosterol and α-tocopherol (contribution 6). Automated flash chromatography separated 15 flavonoids from *Calendula officinalis* with high purity [31].

Such comprehensive profiling illustrates how chemical characterization complements extraction methods, providing insight into both nutritional value and functional potential.

Advanced platforms such as LC-MS/MS, GC-MS, and UV-Vis spectroscopy have further facilitated the identification of flavonoids, terpenoids, and other secondary metabolites in rare or understudied species [32]. These high-resolution techniques reveal structural subtleties that underpin biological activities, including antioxidant and enzyme-modulating effects, which might otherwise go undetected with simpler assays.

Finally, critical reviews highlight the importance of integrating multiple analytical techniques to generate thorough chemical fingerprints of natural products (contribution 7). By combining chromatographic, spectrometric, and spectroscopic data, researchers can detect minor but biologically relevant compounds, strengthen structure–activity correlations, and inform the design of extraction strategies for industrial or nutraceutical applications.

Together, these methodological advances, combining optimized extraction, sustainable protocols, and high-resolution chemical analysis, represent a major step forward in natural product research. They provide a solid foundation for subsequent biological evaluation, functional application, and the translation of plant-derived compounds into therapeutically and nutraceutically relevant products. Pyrolysis–GC/MS analysis characterized the waxy composition of Sicilian propolis, identifying 28 unique biomarkers for geographical authentication [33].

## 4. Biological Evaluation and Functional Activity

This section highlights studies investigating the biological properties of natural extracts and isolated compounds, with particular focus on their mechanisms of action and therapeutic potential.

Citrus-derived compounds, such as naringenin and naringin, have been extensively studied for their anti-inflammatory and protective effects on blood cells. Research demonstrates that these flavonoids can modulate oxidative and inflammatory responses in cellular models, supporting their role as natural anti-inflammatory agents (contribution 3).

Resveratrol from Sicilian pomace inhibits NF-κB in microglial cells, reducing pro-inflammatory cytokines by 65% [34]. Similarly, plant-derived compounds with antioxidant properties have shown promise in mitigating oxidative stress and cellular damage, a key factor in neurodegenerative processes [35].

Saponins and glycosylated triterpenoids from plant sources have revealed notable anticancer potential. For example, engineered glycosylation of specific triterpenoids has yielded novel compounds with enhanced cytotoxic activity against cancer cells, offering new avenues for the development of natural anticancer agents (contribution 7, 8).

Quercetin from Sicilian red onion induces apoptosis in colon cells (HT-29) via p53/Bax upregulation [36].

Marine organisms have also proven to be a rich source of bioactive molecules with antimicrobial activity. Chemical modification of known microbial products has produced derivatives that retain strong antimicrobial efficacy while reducing toxicity, highlighting the potential of marine-derived compounds and their analogs as templates for new antibiotics (contribution 9).

Epigallocatechin gallate (EGCG) from green tea blocks P-glycoprotein, overcoming multi-drug bacterial resistance [37].

Numerous food and plant extracts confirm high antioxidant capacity. Analyses of phenolic acids and flavonoids in hive products such as honey demonstrate significant free radical scavenging activity, emphasizing their potential for functional nutrition and health promotion (contribution 6, 8). Seasonal variation in phenolic profiles has been linked to changes in antioxidant, antihyperglycemic, and anti-inflammatory activity in leaf extracts of certain shrubs, underscoring the complexity and richness of plant-derived antioxidants (contribution 7). Beyond these classical examples, recent studies illustrate the translation of natural compounds into targeted therapeutic applications. Chitosan–oleic acid nanoparticles loaded with lemon peel essential oil have demonstrated bioactive potential for the topical treatment of vulvovaginal candidiasis, while gypensapogenin, a liposome from *Gynostemma pentaphyllum,* efficiently ameliorates hepatocellular lipid accumulation in NAFLD models by activating the FXR receptor, modulating bile acid metabolism (CA/CDCA ratio), and inhibiting CYP7A1/CYP8B1 (contribution 10). These nano-delivery systems enhance the therapeutic efficacy and bioavailability of natural bioactive compounds, exemplifying the integration of natural products with innovative delivery systems (contribution 1). Similarly, investigations on emodin have elucidated both its hepatotoxicity and its potential application in the treatment of liver fibrosis, highlighting the importance of safety evaluation alongside therapeutic exploration (contribution 2).

Together, these studies exemplify the multidimensional nature of natural product research, where chemical characterization, biological validation, and innovative formulation converge to unlock both nutritional and clinical applications. This integrative approach continues to expand the possibilities for developing effective, safe, and sustainable bioactive compounds for human health [38].

## 5. Sustainable Valorization and Technological Applications

This section focuses on studies exploring the sustainable use of natural resources and agro-industrial by-products, highlighting both the environmental benefits and industrial applications.

One key area of research is the valorization of food industry waste for the recovery of phenolic compounds. By applying green solvents and advanced analytical techniques such as GC-MS, researchers have successfully extracted phenolics with high antioxidant potential from agricultural by-products. This approach not only uncovers valuable bioactive compounds but also embodies the principles of a circular economy, turning what was once waste into a resource (contribution 5, 6, 8).

Aromatic plants have also attracted attention for their volatile compound profiles. Using a combination of GC-MS analysis and electronic sensor technologies, scientists have identified the molecules responsible for characteristic aromas. These findings have direct implications for the food and cosmetics industries, where natural fragrances and flavors are increasingly sought-after (contribution 11).

Biotechnological approaches are further expanding the possibilities for sustainable production of bioactive metabolites. Through the use of cell cultures and metabolic engineering techniques, researchers have achieved significant increases in the yield of target compounds. Such strategies offer a pathway to scalable, environmentally friendly production processes, bridging the gap between laboratory research and industrial application (contribution 12, 13).

## 6. Discussion and Future Perspectives

A comprehensive analysis of the contributions published in this Special Issue highlights that research on natural bioactive compounds is rapidly evolving towards an integrated, multidisciplinary model, combining methodological innovations, in-depth biological evaluations, and increasing attention to environmental and social sustainability. However, despite significant progress, important challenges remain that must be addressed to fully translate the potential of these compounds into clinical, nutraceutical, cosmetic, and technological applications.

### 6.1. Remaining Challenges and Critical Issues

One of the main challenges concerns the standardization and validation of analytical and biological methodologies. Currently, variability in extraction, purification, and biological evaluation protocols limits data comparability between studies and slows reproducibility. The intra-species variability of flavonoids in Sicilian sweet basil requires harmonized HPLC-MS protocols for reliable quantification [39].

Therefore, it is essential to develop shared guidelines and harmonized protocols to ensure scientific rigor and reliability, facilitating translation toward industrial and clinical applications (contribution 1, 2).

Blockchain-traceability integration has ensured ISO 22000 compliance for phenolic extracts from olive waste [40,41].

Moreover, although the integration of omics technologies (genomics, metabolomics, proteomics) and artificial intelligence represent a promising frontier for accelerating the discovery and evaluation of bioactive compounds, these approaches are still under development and require substantial investment in infrastructure, training, and interdisciplinary collaboration. The complexity of the generated data also necessitates the development of robust and interpretable predictive models (contribution 3, 6).

Machine learning applied to NMR data predicts the antioxidant activity of plant extracts with 89% accuracy [42].

Another critical point is the need for closer and more structured interdisciplinary collaborations among chemists, biologists, pharmacologists, biotechnologists, agronomists, and clinicians. Only through continuous, integrated dialog will it be possible to develop effective, safe, and sustainable natural products that meet the requirements of increasingly demanding and regulated markets (contribution 7, 8).

Finally, the transition from preclinical research to rigorous clinical studies is an essential step to confirm the efficacy and safety of bioactive compounds in humans. Studies on plant- and marine-derived bioactives have demonstrated promising *in vitro* and cellular effects, highlighting the need for future clinical trials to validate these findings (contribution 10, 12).

### 6.2. Integrated Future Perspectives

Looking ahead, research on natural bioactive compounds is at a stage of great opportunity, but also responsibility. CRISPR/Cas9 editing of the squalene synthase gene in *Nicotiana benthamiana* cells tripled triterpene production [10]. The adoption of standardized and validated methodologies, the integration of omics and artificial intelligence technologies, and the promotion of interdisciplinary collaborations represent the pillars to accelerate the discovery and valorization of new bioactive agents.

At the same time, growing attention to environmental and social sustainability, along with the valorization of biodiversity and the use of green techniques and circular economy principles, will guide the development of natural products that meet the expectations of increasingly quality and environment-conscious markets (contribution 1, 2, 6). Furthermore, strengthening clinical research and translating preclinical findings into concrete therapeutic applications represent the final challenge to bring bioactive compounds from the laboratory to clinical practice, improving human health with natural, safe, and effective solutions (contribution 8, 12, 13). The digital platform “PhytoChain” integrates blockchain, AI, and metabolomics to certify the authenticity of Mediterranean extracts [37].

## 7. Conclusions

In summary, this Special Issue demonstrates the vitality and multidisciplinary nature of research on natural bioactive compounds, highlighting how technological and methodological innovation is opening new avenues for their application in clinical, nutraceutical, cosmetic, and technological fields. However, to fully exploit this potential, current challenges must be overcome, particularly regarding method standardization, integration of emerging technologies, interdisciplinary collaboration, and the conduct of robust clinical trials.

Only through a coordinated effort among researchers, industry, regulatory agencies, and institutions will it be possible to transform nature’s richness into innovative, sustainable solutions that provide real benefits for society and the environment.

## Abbreviations

The following abbreviations are used in this manuscript:

CBGA	Cannabigerolic Acid
CRISPR	Clustered Regularly Interspaced Short Palindromic Repeats
DPPH	2,2-Diphenyl-1-picrylhydrazyl
GC-MS	Gas Chromatography–Mass Spectrometry
HPLC	High-Performance Liquid Chromatography
HRMS	High-Resolution Mass Spectrometry
LC-MS/MS	Liquid Chromatography–Tandem Mass Spectrometry
NMR	Nuclear Magnetic Resonance
Omics	Genomics, Proteomics, Metabolomics
ROS	Reactive Oxygen Species
UV-Vis	Ultraviolet–Visible Spectroscopy
